# Synthesis of Marine Polycyclic Polyethers *via Endo*-Selective Epoxide-Opening Cascades

**DOI:** 10.3390/md8030763

**Published:** 2010-03-19

**Authors:** Ivan Vilotijevic, Timothy F. Jamison

**Affiliations:** Department of Chemistry, Massachusetts Institute of Technology, 77 Massachusetts Avenue, Cambridge, MA 02139, USA; E-Mail: vilotije@mit.edu (I.V.)

**Keywords:** marine polyethers, epoxide-opening cascades, biomimetic synthesis

## Abstract

The proposed biosynthetic pathways to ladder polyethers of polyketide origin and oxasqualenoids of terpenoid origin share a dramatic epoxide-opening cascade as a key step. Polycyclic structures generated in these biosynthetic pathways display biological effects ranging from potentially therapeutic properties to extreme lethality. Much of the structural complexity of ladder polyether and oxasqualenoid natural products can be traced to these hypothesized cascades. In this review we summarize how such epoxide-opening cascade reactions have been used in the synthesis of ladder polyethers and oxasqualenoid natural products.

## 1. Introduction

Marine polyether natural products, polycyclic molecules of marine origin with regular occurrence of multiple C-O-C motifs, include the distinct families of the ladder polyethers and the oxasqualenoids. Members in both of these families exhibit biological activities ranging from potentially therapeutic properties to extreme lethality. They have received attention from diverse scientific communities owing to their involvement in harmful algal blooms, their devastating effects on marine ecosystems and the industries that depend on their continued health, their impact on public health in coastal areas, their beneficial properties that make them useful probes of protein structure and function, and, in recent years, to their potential to treat the symptoms of cystic fibrosis.

**Figure 1:**
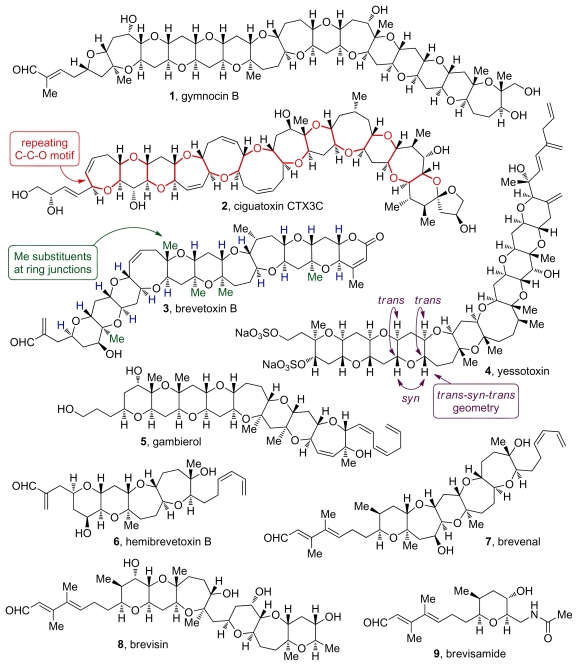
Representative ladder polyether natural products.

**Figure 2:**
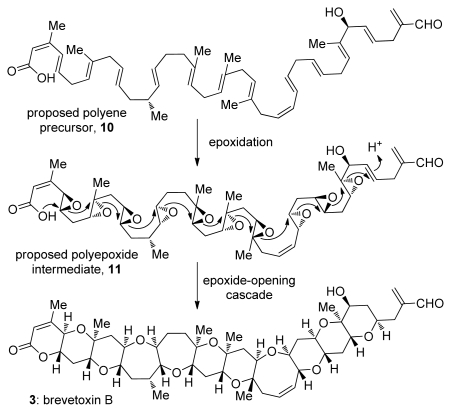
Nakanishi hypothesis: a model of brevetoxin B biosynthesis *via* an epoxide-opening cascade.

**Figure 3:**
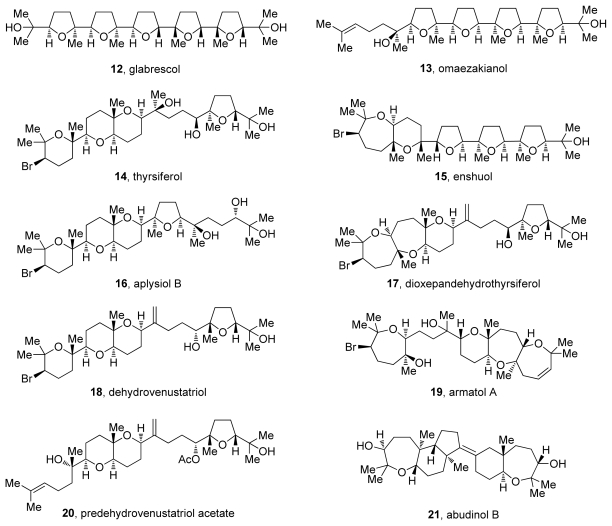
Representative oxasqualenoid natural products.

The proposed biosynthetic pathways to both ladder polyethers of polyketide origin and oxasqualenoids of terpenoid origin share dramatic epoxide-opening cascades as key steps [1,2]. These reactions are not only postulated to be involved in the biogenesis of polyethers, but also could be utilized as a method to rapidly construct the molecular frameworks in these natural products [3]. Much of the structural complexity of ladder polyether and oxasqualenoid natural products can be traced to these hypothesized cascades. The first group, ladder polyethers (Figure 1), includes molecules with multiple fused cyclic ethers that are proposed to be formed *via* all-*endo* cascades of epoxide openings (Figure 2). The other group consists of molecules that are produced *via* cascades that feature *exo*- and/or *endo*-selective epoxide-opening reactions. In this class, the cyclic ethers are either connected by a carbon-carbon bond or fused to each other (Figure 3). Our discussions on oxasqualenoid natural products, however, will be limited to natural products from this family that resemble ladder polyethers and feature multiple fused rings; that is, to those members of the family that are proposed to originate from biosynthetic cascades with multiple *endo*-selective epoxide-opening steps.

In this review we provide a summary of various approaches to achieving *endo*-selective intramolecular epoxide-opening reactions, strategies to extend these reactions into epoxide-opening cascades (more than one epoxide opening), and the application of these reactions to the synthesis of ladder polyethers and oxasqualenoids. The material is organized by the size of the rings that are formed in epoxide-opening cyclizations, tetrahydropyrans and oxepanes. A further distinction is made between the approaches developed for control of regioselectivity in epoxide-opening reactions, *i.e.*, those that rely on electronically biased epoxides and those in which the epoxides may be considered to be electronically unbiased.

## 2. Ladder Polyethers

The ladder polyether natural products feature five- to nine-membered cyclic ethers fused to each other in a *trans*-*syn*-*trans* arrangement (Figure 1). The smallest member of the family, hemibrevetoxin B (**6**, Figure 1), contains only 4 rings while the largest, maitotoxin, features four polyether ladders with an unequaled total of 32 rings. It is thus the largest, fully characterized, nonpolymeric structure isolated from natural sources [4,5]. Ring junction substituents of ladder polyethers are normally hydrogen atoms, and at some ring junctions, one of the H atoms is replaced with a methyl group. A repeating C-C-O sequence can be traced from one end to the other in the polycyclic core of these molecules. The recent isolation of brevisamide (**9**, Figure 1), a member of this family that features a single cyclic ether, and brevisin (**8**, Figure 1), an aberrant polycyclic polyether with two distinct ring systems, challenge some of the previous notions on structure of ladder polyethers and underline the remarkable diversity of polyether structures found in nature.

The first reported member of this family, brevetoxin B, was isolated and characterized by Nakanishi and Clardy in 1981 [6]. Subsequent studies have revealed the structures of numerous ladder polyethers, reshaped the boundaries in natural products chemistry, and pushed the limits of the analytical methods used in structure determination of organic molecules [7–9]. Their structures continue to inspire synthetic chemists to develop new synthetic methods [10–13].

Ladder polyethers are perhaps best known for their association with red tides, harmful algal blooms that involve a rapid increase in concentration of algae in coastal areas [14]. Some of the ladder polyether-producing dinoflagellates, such as *Karenia brevis*, participate in these blooms, causing devastating killings of fish and marine mammals. Marine species that are not affected by red tide toxins accumulate and further elaborate the toxins [15,16]. In this way, the toxins move up the food chain causing occasional poisoning in humans [17].

While their structures appear uniform, ladder polyethers exhibit diverse biological activities. Many members of this family bind sodium [18], potassium [19] and calcium [20] ion channels with high affinity, thus disrupting the function of these proteins. Ladder polyethers can also show beneficial anti-cancer [21,22] and antifungal [23,24] properties. Notably, brevenal is a lead compound for the development of treatment for cystic fibrosis and neurotoxic shellfish poisoning [25–27].

The biosynthesis of ladder polyethers became a popular topic of speculation soon after the structure of brevetoxin B was determined. The polyether ionophores, a related group of polycyclic natural products featuring smaller rings, had already been extensively studied by this time [28], allowing the comparison of the newly isolated structures to well known natural products such as nigericin, lasalocid and monensin A. The analysis of polycyclic structures of various polyether ionophores featuring multiple five and six membered cyclic ethers interconnected *via* carbon-carbon bonds led to a unified biosynthetic proposal for these natural products [29]. Soon after Cane, Celmer and Westley proposed that the *Streptomyces* bacteria, organisms that produce various polyether ionophores do so *via* all-*exo* epoxide-opening cascade reactions, similar structural analysis and biosynthetic proposals were described for ladder polyethers [30]. Two structural features of ladder polyethers were particularly important for the biosynthetic proposal that is often referred to as Nakanishi’s hypothesis: the repeating C-C-O motif that spans the whole ladder polyether molecule and the uniform *trans*-*syn*-*trans* geometry of the ring junctions. Nakanishi and Shimizu independently hypothesized that the structural and stereochemical uniformity of ladder polyethers arise through the transformation of a polyepoxide into a ladder polyether *via* a series or cascade of epoxide-opening events [31–33] (Figure 2). The oxygen and two carbon atoms of each epoxide constitute the C-C-O backbone of a ladder polyether. The *trans*-*syn* topography is also explained by this mechanism provided that inversion of configuration occurs during each epoxide opening, and that all epoxides are derived from *E* alkenes. As was recently discussed by Spencer, all alkenes in a hypothetical precursor would require the identical stereoselectivity of epoxidation, producing either an all-(*S*,*S*) or all-(*R*,*R*) polyepoxide. It was consequently suggested that a single promiscuous monooxygenase might be sufficient for all alkene to epoxide oxidations in the biosynthesis of a ladder polyether [34].

Analysis of the Cane-Celmer-Westley and Nakanishi hypotheses in light of Baldwin’s rules of ring closure (see Chapter 4) reveals a potential pitfall of the latter. In general, intramolecular epoxide-opening reactions preferentially produce the smaller heterocycle e.g., THF instead of THP, *via* an *exo*-pathway [35] (Figure 5b). While most of the epoxide-opening steps in the Cane-Celmer-Westley proposal proceed *via* the favored *exo*-pathway, Nakanishi’s hypothesis relies upon a ring-opening process generally considered to be disfavored. Thus, an epoxide-opening cascade to produce brevetoxin B must overcome ten separate disfavored epoxide openings (Figure 2). Epoxide hydrolase enzymes would likely control such disfavored process in the producing organisms. This regiochemical problem, however, represents a major obstacle for attempts to emulate such epoxide-opening cascades in the synthesis of polyether ladders.

Labeling studies for brevetoxin A [32], brevetoxin B [31,33] and yessotoxin [36] together with recent genetic analyses [37–39] have provided some insight into their polyketide origin. However, these studies failed to shed light on the proposed subsequent epoxidation and cyclization steps. Remote evidence in support of Nakanishi’s hypothesis can be taken from the biosynthetic studies on okadaic acid [40,41]. These studies demonstrated that the oxygen atom incorporated at the fused THP diad of okadaic acid is derived from molecular oxygen, suggesting the involvement of an epoxide intermediate in the formation of this ladder polyether-like motif. The isolation of the epoxy analogue of brevetoxin B, 27,28-epoxybrevetoxin B, may also suggest involvement of epoxides in the biosynthetic route to ladder polyethers [42]. Finally, the recently disclosed atypical structures of brevisamide and an aberrant polyether, brevisine may help further the understanding of ladder polyether biosynthesis [43,44].

Although Nakanishi’s proposal remains speculative, it has served as the basis for a structural revision of brevenal [45,46], and a proposed structural revision of maitotoxin [34,47,48]. It also remains an inspiration for numerous cascade approaches for the rapid synthesis of ladder polyether natural products.

A noteworthy extension of Nakanishi’s proposal stems from the analysis of stereochemical uniformity in ladder polyethers. The Spencer group suggests that a polyepoxide intermediate may not be required in the biosynthesis of ladder polyethers from proposed polyene precursors. Instead, it was proposed that this transformation can be achieved *via* iterative action of a monooxygenase and an epoxide hydrolase both with broad specificity [34].

In addition to Nakanishi’s proposal, two different biosynthetic hypotheses that involve transformation of an all-*Z* polyene precursor to a ladder polyether molecule have been proposed. The first proposal includes a series of oxidative cyclizations of an all-*Z* polyene precursor that would presumably proceed *via* a [2 + 2] mechanism and would involve the action of a Fe-containing mono-oxygenase enzyme [49–51]. The second proposal involves a series of rearrangements of *cis*-epoxy esters in which both epoxide carbon atoms undergo inversion of configuration to produce the *trans-*fused junction of a ladder polyether [52,53]. Neither of these proposals has been experimentally evaluated in the context of the biosynthesis of ladder polyethers.

## 3. Oxasqualenoids, Polyethers Derived from Squalene

Oxasqualenoid natural products come from diverse sources including tropical plants, marine sponges and red algae. These polycyclic polyethers derived from squalene often resemble polyether ionophores due to the regular occurrence of tetrahydrofuran rings interconnected by carbon-carbon bonds (e.g., omaezakianol and enshuol, Figure 3). As noted earlier, our discussion on oxasqualenoids will be limited to those members of the group that feature multiple fused rings, exemplified by the skeletons of dioxepandehydrothyrsiferol and armatol A (**17** and **19**, Figure 3). Such oxasqualenoids are isolated only from marine sources, most of them from red algae of the *Laurencia* and *Chrondria* genera [2]. In addition to these highly oxygenated triterpenes, squalene-derived polycycles like abudinol B (**21**, Figure 3) have also been isolated from marine sponges. These metabolites typically feature two separate cyclic systems and contain up to two cyclic ethers that are not fused to each other.

Most reports of isolation of new members of this family are regularly accompanied by a biosynthetic proposal for the specific carbon skeleton [54–57]. The common ground for all of the proposals is the involvement of epoxide-opening cascades [2,58,59]. Biosynthetic studies on these molecules are, unfortunately, scarce. Their triterpene origin is rather obvious as there are typically no skeletal rearrangements that can be detected in oxasqualenoid molecules. However, subsequent oxidation and cyclization steps remain speculative.

If the proposed cascades in fact do occur in nature, the diverse geometry of the ring junctions of oxasqualenoids (e.g., *trans*-*syn*-*trans* in armatol A and *trans*-*anti*-*trans* in dioxepandehydrothyrsiferol) can either be established by the stereospecific transformation of appropriately configured epoxides in the polyepoxide precursor or by pathways that involve diversification brought *via* non-stereospecific transformations of the polyepoxy precursors. Over the years a number of structures consistent with the mentioned proposals have been isolated, thus contributing to their credibility.

**Figure 4:**
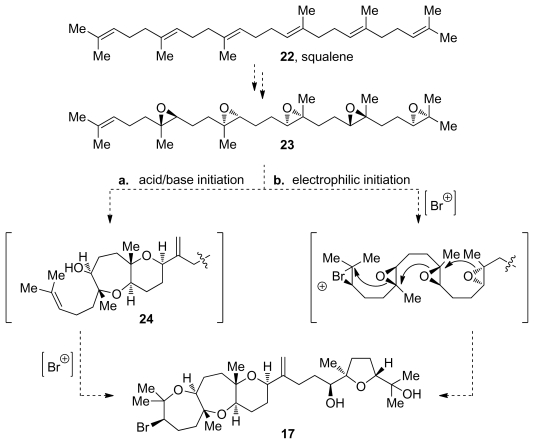
Proposed biosynthetic pathways for the dioxepandehydrothyrsiferol tricycle: (**a**) epoxide-opening cascade followed by bromoetherification. (**b**) epoxide-opening cascade initiated by bromonium formation.

The presence of a halide in positions 2 or 3 of the oxasqualenoid molecules isolated from red algae is an interesting structural feature that has itself drawn biosynthetic speculation. Isolation of predehydrovenustatriol acetate (**20**, Figure 3) [57], a molecule that lacks the halogenated ring but otherwise features fully cyclized structure of dehydrovenustatriol (**18**, Figure 3), which is isolated from the same source, suggests that at least two discrete biosynthetic steps are involved in the cyclization of the polyepoxy precursor. In this scenario, the halide is installed *via* haloetherification of the alcohol intermediate **24** produced in the epoxide-opening steps that are presumably achieved *via* acid-base catalysis (pathway a., Figure 4). However, formation of a bromonium species by action of an electrophilic agent could also initiate the epoxide-opening cascade reaction, thus producing the complete polycycle in a single step (pathway b., Figure 4) [60].

## 4. Baldwin’s Rules

**Figure 5:**
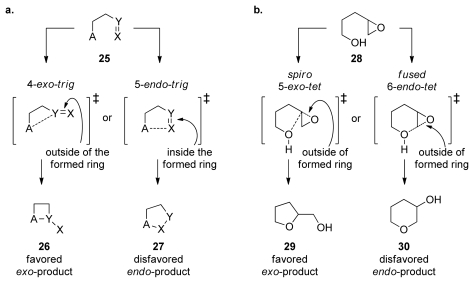
Baldwin’s rules: (**a**) general classification. (**b**) as they apply to intramolecular epoxide opening.

Regio- and stereoselectivity in epoxide-opening reactions are of great importance in the context of epoxide-opening cascades as they determine the composition of the final reaction products. Baldwin’s rules of ring closure, empirical guidelines for evaluation of ring forming processes, are generally used to predict the outcome of ring-forming reactions, including the intramolecular epoxide openings. The size of the formed ring, the position of the bond that is broken relative to the smallest formed ring, and the geometry of the electrophile are the criteria used to classify ring-forming reactions [35]. If the position of the bond broken during the reaction is outside of the newly formed ring, then the reaction is classified as *exo*. If the broken bond is within the smallest formed ring, the reaction is classified as *endo* (Figure 5a). In Baldwin’s classification, reactions involving *sp^3^* hybridized electrophiles are described as *tet* due to the tetragonal geometry of the electrophile (*sp^2^* hybridized electrophiles are *trig*, and *sp* electrophiles are digonal or *dig*). Based on this classification, Baldwin formulated a simple set of guidelines to predict the relative feasibility of different ring-closing reactions. Although empirical, these rules are based on stereoelectronic considerations [61]. The favored ring-closing reactions are those in which the length and nature of the linking chain enable the terminal atoms to achieve the proper geometries for the reaction. The disfavored ring closing processes require distortions of bond angles and bond distances rendering these reaction pathways higher in energy. For instance, 5-*endo*-trig ring closing reactions are predicted to be disfavored over 4-*exo*-trig reactions (Figure 5a).

Baldwin’s rules were not specifically formulated for epoxide-opening reactions. However, intramolecular epoxide openings tend to follow rules that lie between those for tetrahedral and trigonal systems, generally favoring the *exo* processes, which proceed *via* spiro transition state (Figure 5b). Intramolecular epoxide-opening reactions, with few exceptions, favor the smaller over the larger heterocycle (e.g., tetrahydrofuran **29**, produced *via* a spiro transition state over the tetrahydropyran **30**, from fused transition state, Figure 5b). Baldwin’s rules classify the fused and spiro transition states as *endo* and *exo*, respectively. However, because the epoxide C-O bond that breaks is outside the newly formed ring in both cases, each may also be considered to be an *exo* process under the same construct (Figure 5b). To avoid potential confusion, we prefer the terms “fused” and “spiro” that describe transition states leading to *endo* and *exo* products respectively, by analogy to the reactions of cyclopropanes [62].

Epoxides have long been regarded to be useful and highly versatile intermediates in organic synthesis and many efficient methods for enantioselective epoxidation have been developed. The Sharpless asymmetric epoxidation of allylic and homoallylic alcohols [63–65], Jacobsen epoxidation [66–69] and the Shi epoxidation of unactivated alkenes [70–73] in particular, represent irreplaceable tools for investigations of epoxide-opening cascades. These methods enabled efficient syntheses of many of the epoxides that will be discussed herein, thus catalyzing explorations in the field of epoxide-opening cascades.

Effective ways to control the regioselectivity in epoxide-opening reactions are necessary for epoxides to be used as versatile intermediates in synthesis. Since the *exo* mode of cyclization is typically preferred, methods to facilitate *endo* selective cyclization have constituted a particularly active area of research.

## 5. Construction of Tetrahydropyrans *via Endo*-Selective Epoxide-Opening Reactions

Most of the approaches to promote the desired, *endo* outcome of intramolecular epoxide openings rely on the effects of directing groups covalently attached to the epoxides. When chosen and positioned properly, the epoxide substituents provide an electronic bias, favoring *endo* cyclization and formation of the larger ring. These directing groups act either by stabilizing the desired transition state or, alternatively, by slowing the undesired cyclization route, thus promoting regioselective nucleophilic attack.

### 5.1. Reactions of electronically biased epoxides

#### 5.1.1. Alkenylepoxides

**Scheme 1:**
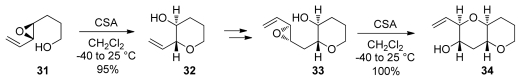
Activation of the 6-*endo* over the 5-*exo* epoxide-opening pathway: iterative approach to oligotetrahydropyrans [75]. CSA = 10-camphorsulfonic acid.

Pioneering studies on the activation of the 6-*endo* over the 5-*exo* epoxide-opening pathway in intramolecular reactions of 4,5-epoxy alcohols were reported by Nicolaou [74]. The epoxy alcohol **31** bearing an alkenyl directing group undergoes Brønsted acid-catalyzed cyclization with excellent *endo* selectivity due to the ability of the alkenyl substituent to stabilize the partial positive charge in the transition state for *endo* cyclization (Scheme 1). These reactions established the first well-defined and predictable routes to tetrahydropyran systems *via* 6-*endo* epoxide opening.

Additional flexibility in this methodology comes from the opportunity to fine tune the reactivity of the epoxide by changing the electronic characteristics of the alkenyl group (*i.e.,* unsaturated ester *vs.* vinyl halide) [75]. The fact that disubstituted epoxides are generally considered to be good substrates in these reactions together with various options for further elaboration of the alkenyl substituents of the reaction products made this approach a standard tool for the construction of tetrahydropyran rings of ladder polyethers. It has been utilized by the groups of Nicolaou, Yamamoto, Nakata, Mori, and Sasaki in their syntheses of hemibrevetoxin [76–79], brevetoxin B [80–87], brevetoxin A [88–92], gambierol [93–96], and brevenal [45,46].

**Scheme 2:**
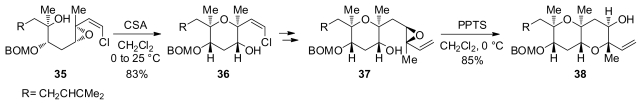
Iterative syntheses of the FG fragment of brevetoxin B [97]. BOM = benzyloxymethyl; PPTS = pyridinium *p*-toluenesulfonate.

Although not amenable to more than one epoxide-opening at a time, this approach was instrumental in various iterative syntheses of ladder polyethers and their fragments. The Nicolaou group utilized this methodology to prepare the FG fragment of brevetoxin B during the first total synthesis of a ladder polyether natural product [97]. Nicolaou’s approach includes sequential acid-catalyzed openings of alkenyl epoxides to form both the F and G rings of brevetoxin B (Scheme 2). Epoxy alcohol **35** was efficiently transformed to a corresponding tetrahydropyran **36** which could then be elaborated to **37**. Subsequent Brønsted acid-catalyzed epoxide opening of **37** affords **38**, containing the F and G rings of brevetoxin B.

#### 5.1.2. α,β-Epoxysulfones

**Scheme 3:**
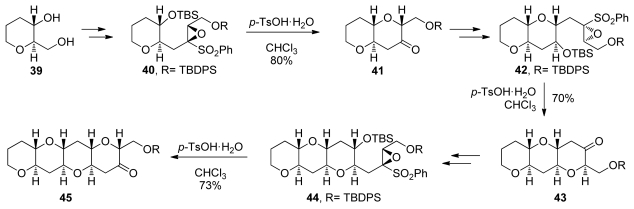
Synthesis of oligotetrahydropyran fragments *via* iterative 6-*endo* cyclizations of epoxysulfones [98]. TBS = *tert*-butyldimethylsilyl; Ts = tosyl; TBDPS = *tert*-butyldiphenylsilyl.

A conceptually orthogonal approach to the construction of tetrahydropyran rings *via* 6-*endo* intramolecular epoxide-openings was reported by Mori. Instead of activating the 6-*endo* pathway, the Mori group use sulfonyl substituents on the epoxide to destabilize the transition state that would lead to 5-*exo* epoxide opening [98]. Exposure of epoxysulfone **40** to Brønsted acids affords ketone **41** *via* 6-*endo* cyclization and subsequent loss of phenylsulfonate (Scheme 3). A sequence involving alkylation of the sulfone-stabilized *cis*-oxyranyl anion completes the homologation process to **42**, the next epoxysulfone primed for *endo* selective cyclization. Repeating this procedure three times leads to formation of tetrad **45**. An obvious limitation of this approach is the inability to incorporate substituents other than sulfonyl group at the position of 5-*exo* attack. This may be circumvented *via* the addition of organomagnesium reagents to the ketone formed after cyclization, a reaction that typically proceeds with good stereoselectivity to ultimately produce fully substituted *trans*-fused fragments of ladder polyethers [99].

The tetrahydropyranones produced in reactions developed by the Mori group are amenable to a single-carbon homologation effected by trimethylsilyldiazomethane to produce ring expanded, 7-membered cyclic ethers [100]. Mori and coworkers took advantage of this feature in the total syntheses of hemibrevetoxin B [79,101], gambierol [96], and in the iterative synthesis of the ABCDEF ring system of the yessotoxins and adriatoxins (**4**, Figure 1) [102]. Notably, the same epoxysulfone **50** was used for the construction of four of the six rings in Mori’s synthesis of ABCDEF fragment of yessotoxin and adriatoxin. To introduce methyl substituents at the ring junctions of **46** efficiently, Mori has developed two strategies [103]. The first requires elaboration of a 3-ketooxepane to a corresponding 3-methylideneoxepane, followed by epoxidation and reduction of the epoxide with lithium triethylborohydride. The other method is the inherently more convergent incorporation of a methyl substituent into the epoxysulfones **48** and **51** that installs the two remaining methyl groups.

#### 5.1.3. Methoxymethyl substituted epoxides

**Scheme 4:**
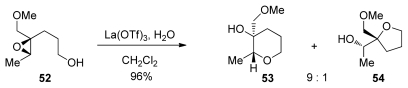
Methoxymethyl directed *endo*-selective epoxide-opening reactions [104].

The Murai group has reported a cascade approach to the synthesis of ladder polyethers involving *endo*-selective, lanthanide-promoted opening of epoxides bearing methoxymethyl groups. When epoxy alcohol **52** is treated with La(OTf)_3_ in the presence of 2.2 equivalents of water in dichloromethane, it undergoes a clean cyclization to produce predominantly the 6-membered ether **53** with 9:1 selectivity (Scheme 4) [104,105]. The methoxymethyl group may exert a similar electronic bias as the sulfonyl group, as it inductively destabilizes positive charge at the 5-*exo* position. However, these reactions require a unique set of conditions to proceed in good selectivity, suggesting that the lanthanide Lewis acid may chelate the epoxide oxygen and the oxygen in the directing group, thus providing geometrical constrains that are responsible for the observed selectivities.

**Scheme 5:**
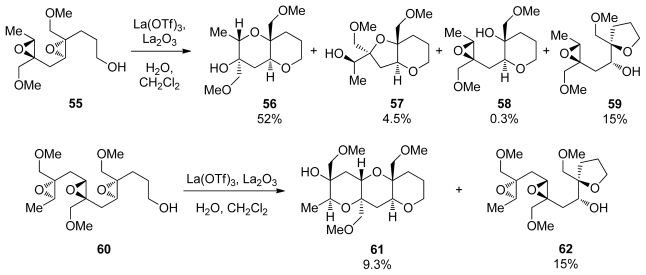
Epoxide-opening cascades of methoxymethyl substituted epoxides catalyzed by lanthanum-based Lewis acids [106].

Murai and coworkers also prepared polyepoxides **55** and **60**, which contain a methoxymethyl directing group at each epoxide. When treated with La(OTf)_3_ diepoxide **55** was converted into the THP diad **56** with methoxymethyl groups present at the ring junctions (Scheme 5) [106]. The side products isolated in this reaction suggest a pathway that proceeds in a stepwise fashion from the primary alcohol nucleophile initially forming intermediate **55**. The intermediate epoxyalcohol subsequently undergoes further cyclization to afford **56** and **57**. Murai has also demonstrated that cascades directed by methoxymethyl groups in combination with an appropriate chelating Lewis acid can be extended to provide larger ladder polyether-type fragments, such as triad **61**, albeit in low yield.

#### 5.1.4. Trialkyl substituted epoxides

With the exception of the methoxymethyl-directed reactions, the *endo*-selective epoxide-opening reactions described so far are not amenable to cyclizations of more than one epoxide. Although these approaches can be quite useful in preparing tetrahydropyrans in various contexts in natural product synthesis, the products of these reactions require additional elaboration into naturally occurring structures *via* either removal of the directing group or elaboration of the functional groups present in the final product into the fragments of the naturally occurring compounds. These problems arise because the directing groups used to secure good *endo* selectivity are not present in the natural compounds. Unlike vinyl, sulfonyl or methoxymethyl groups, methyl groups often substitute hydrogens at ring junctions of both ladder polyethers and oxasqualenoids and thus can be utilized to direct regioselectivity of epoxide opening without the need for their removal from the final product of such reactions. The ability of methyl groups to stabilize positive charge in the intermediates of epoxide-opening reactions with trialkyl epoxides is well precedented [3], and the first systematic reports on the directing effects of methyl groups attached to the epoxide were reported recently by the McDonald group [107].

The McDonald group explored epoxide-opening cascades for the synthesis of polytetrahydropyrans from polyepoxy substrates featuring appropriately positioned methyl substituents at each of the epoxides [107] (Table 1). The methyl groups are placed strategically at the carbon atoms expected to undergo nucleophilic attack in the *endo* transition states so that they can stabilize the positive charge developed in the transition state of these acid-catalyzed reactions. They discovered that the nature of the terminating nucleophile plays an important role in these reactions as demonstrated in the epoxide-opening cascades of 1,4-diepoxides **63**–**67**. These reactions can proceed with either retention or inversion of configuration at the ring junction depending on the type of pendent nucleophile. Stronger nucleophiles at elevated temperatures tend to favor the clean inversion of stereochemistry in the opening of the internal epoxide, affording the *trans*-fused **68**. In contrast, less nucleophilic carbonates favor the production of diastereomeric *cis*-fused product **69**, corresponding to retention of configuration.

**Scheme 6:**
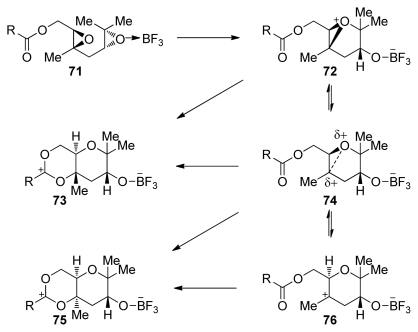
Mechanistic rationale for the effects of the type of terminating nucleophile on the stereochemical outcome of epoxide-opening cascades [107].

The mechanism in which the terminating nucleophile intercepts a cationic intermediate at different points in the continuum between the extremes of epoxonium ion **72** and tertiary alkyl carbocation **76** explains these observations (Scheme 6). The McDonald group proposed that *cis-*fused products arise from fast nucleophilic addition to the tertiary carbocation, whereas *trans-*fused products are favored with a stronger nucleophile, which intercepts a tight ion pair intermediate structurally related to the epoxonium ion [107].

**Scheme 7:**
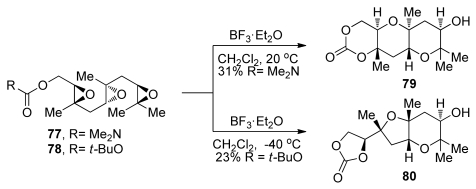
Effects of the terminating nucleophile on the outcome of a methyl-directed cascade leading to polytetrahydropyran systems [107].

Similar trends were observed in the more challenging epoxide-opening cascades of triepoxides **77** and **78**, which feature a methyl directing group on each of the epoxides [107]. When activated by a Lewis acid at an appropriate temperature, triepoxide **77**, featuring a stronger, carbamate terminating nucleophile, is transformed into the ladder polyether-like tricycle **79** in 31% yield (Scheme 7). In contrast, triepoxy carbonate **78** failed to afford any of the desired product and instead gave a fused THF/THP product **80** at low temperatures. Product **80** is presumably produced through isomerization of the initially formed bicyclo[3.1.0]epoxonium intermediate, which leads to *cis* geometry at the ring junction, followed by 5-*exo* cyclization in the last epoxonium opening event of the cascade. The McDonald group also investigated a variety of conditions for the activation of the epoxide and demonstrated that, in some cases, the choice of Lewis acid may determine the final outcome of related epoxide-opening cascades.

**Scheme 8:**
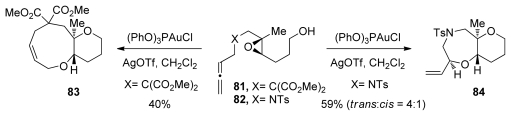
Gold(I)-catalyzed cascade cyclization of allenyl epoxides [108].

Gagné and coworkers recently reported cascades that involve epoxide-opening in which the regioselectivity is controlled by the strategic incorporation of methyl substituents [108]. To initiate the cyclization reaction, a cationic gold(I) phosphite catalysts is used to activate the allenes of **81** and **82** to form epoxonium intermediates that then undergo nucleophilic attack by the pendant alcohol (Scheme 8). By stabilizing the intermediary carbocation character, the methyl substituent serves as a directing group for nucleophilic attack. Substrates that feature more than one epoxide were not described in this study. Nonetheless, these experiments demonstrate the diversity of the initiating conditions in epoxide-opening cascades.

**Scheme 9:**
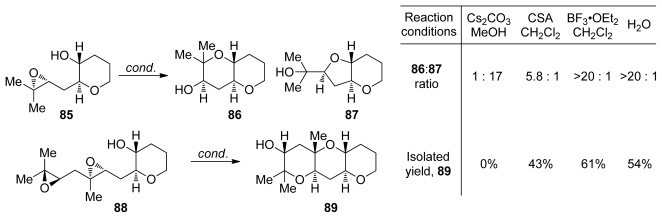
Methyl-directed epoxide openings in water [111].

The effect of reaction conditions on the outcome of epoxide-opening cascades of 1,4-diepoxides in which each of the epoxides features a methyl substituent at the position of 6-*endo* opening were recently evaluated by the Jamison group. These studies were carried out as further investigation of the aqueous, templated epoxide-opening cascade reactions developed in the Jamison group [109] (*vide supra*, Chapter 5.2). As expected, acid-catalyzed reaction of **85** afforded predominantly the *endo*-product **86**, mirroring the previous observations regarding the selectivity of epoxide-opening reactions of trisubstituted epoxides (Scheme 9). Under basic conditions, however, the *exo*-product predominates with selectivity of 17:1. The best promoter for these reactions proved to be deionized water. Aqueous reactions provide the desired *endo*-product **86** in high yield and with good selectivity owing to the suppression of side reactions common in Lewis acid-catalyzed reactions, such as, the rearrangement of epoxides to corresponding ketones *via* 1,2-hydride shift. Cascades of diepoxide **88** follow the same trend with good *endo* selectivities leading to the formation of **89** observed in water and under acidic conditions [110,111].

**Scheme 10:**
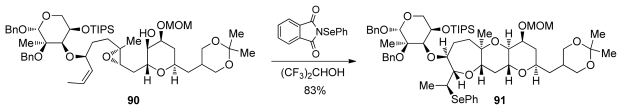
Application of electrophile-initiated epoxide-opening cascade in the synthesis of hemibrevetoxin B [112]. TIPS = triisopropylsilyl; MOM = methoxymethyl.

An epoxide-opening cascade that capitalizes upon the presence of a methyl group in an appropriate position was utilized by Holton and coworkers in the total synthesis of hemibrevetoxin B [112]. Although only one epoxide is involved in this reaction, two cyclic ethers of the natural product are produced in a single operation. Activation of the Z-alkene in **90** with *N*-(phenylseleno)phthalimide *via* the formation of a selenonium ion sets the stage for *exo* opening by the epoxide nucleophile and formation of an epoxonium intermediate (Scheme 10). The *endo* cyclization onto the epoxonium intermediate directed by the methyl substituent produces **91**, which contains the *trans*-fused BC ring system of hemibrevetoxin B (**6**, Figure 1). Use of a highly polar, non-nucleophilic solvent such as, hexafluoro-*iso*-propanol increases the selectivity by enabling a higher degree of charge separation in the transition state. According to computational studies by Houk, the existence of this loose, S_N_1-like transition state is required in alkyl group-directed 6-*endo* cyclizations [113–115].

**Scheme 11:**
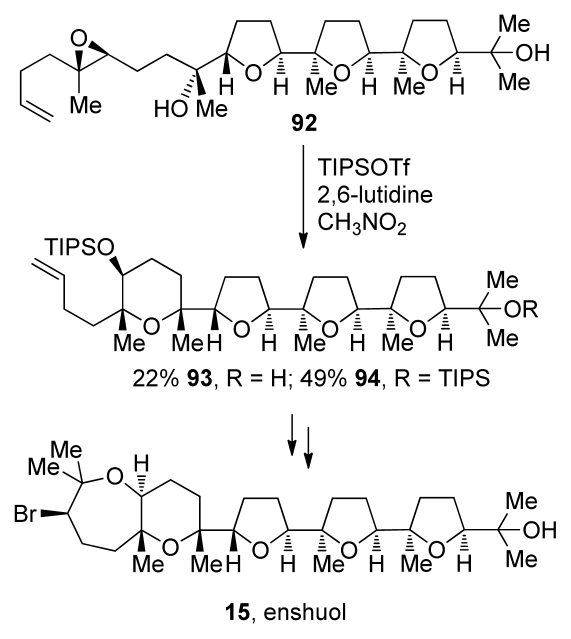
*Endo*-selective epoxide opening of a trisubstituted epoxide with a tertiary alcohol: application to the synthesis of enshuol [117].

Morimoto and coworkers also took advantage of the directing effect of methyl groups [116]. They use methyl substituents to increase *exo* selectivity in epoxide-opening cascades that lead to polytetrahydrofuran segments of oxasqualenoids but also, when appropriately positioned, to promote *endo* selectivity in construction of the THP rings in the total synthesis of enshuol [117] and aurilol [118]. When treated with TIPSOTf, epoxy alcohol **92** undergoes cyclization to form the mixture of **93** and **94**, the penultimate precursor to enshuol. These reactions require a tertiary nucleophile and a bulky silyl triflate to avoid side reactions such as conversion of the alcohol nucleophile to the silyl ether (Scheme 11).

As demonstrated by the groups of McDonald [107], Jamison [111], Holton [112] and Morimoto [116–118], methyl substituents are reliable directing groups in epoxide-opening cascades leading to THPs. However, successful cascades usually require appropriately positioned methyl substituent at each of the epoxides. The products obtained this way contain methyl substituents uniformly distributed at ring junctions across the polyether ladder. Although methyl groups are present at the ring junctions of ladder polyethers, they are normally arranged in a random fashion, and generally appear at no more than half of the ring junctions. Hence, fragments produced *via* methyl group-directed epoxide-opening cascades rarely appear in natural products. Methods that can produce polytetrahydropyrans with no substituents (*i.e.*, only H atoms) at the ring junctions further expand the utility of epoxide-opening cascades in the synthesis of naturally occurring polytetrahydropyran fragments.

#### 5.1.5. Epoxysilanes

**Scheme 12:**
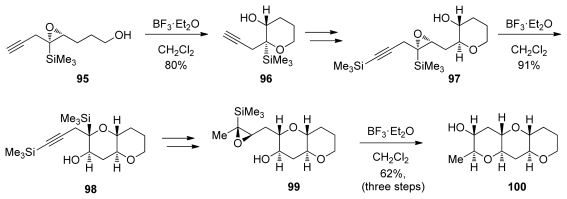
6-*endo* cyclization of epoxysilanes: iterative approach to the synthesis of oligotetrahydropyrans [124].

Similar to alkyl substituents, trialkylsilyl groups also have the potential to stabilize positive charge in the transition state leading to the 6-*endo* opening of epoxides. The structural effect of a silyl group attached directly to an epoxide has been studied in detail by Hudrlik [119–122] and Paquette [123]. These results have found application in the *endo*-selective intramolecular reactions of epoxysilanes developed in the Jamison group [124]. Lewis acid-catalyzed *endo*-selective intramolecular epoxide-opening reactions of epoxysilanes were utilized in iterative syntheses of *trans-*fused oligotetrahydropyran fragments (Scheme 12). Conveniently, after cyclization, removal of the directing group can be achieved cleanly with TBAF *via* a Brook rearrangement. This enables the synthesis of fragments that do not contain directing groups in the polyether ladders, as demonstrated by synthesis of the THP triad **100**.

**Scheme 13:**
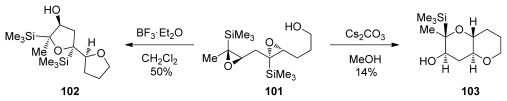
Silyl-directed cascade cyclizations of epoxysilanes [125].

When extended to diepoxides, the Lewis acid-catalyzed epoxide opening reactions of epoxysilanes fail to provide the desired product. Despite the suitably positioned directing groups, when treated with Lewis acids, diepoxide **101** yields bistetrahydrofuran **102** as the only isolable product (Scheme 13). Thorough evaluation of reaction conditions revealed that the outcome of this reaction was very different when a Brønsted base in alcoholic solvents was used instead (Scheme 13) [125]. Under these conditions diepoxide **101** undergoes a cascade reaction to produce THP diad **103**. It was noted in this transformation that the trimethylsilyl directing group was fortuitously absent from the ring junction in the product.

**Scheme 14:**
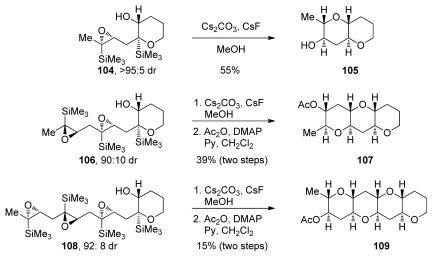
Epoxide-opening cascades with a disappearing silyl group [125].

Further modifications to the design of polyepoxide substrates and reaction conditions resulted in the development of epoxide-opening cascades directed by “disappearing” silyl groups (Scheme 14) [125]. These modifications include the construction of one THP ring (as in **104**, **106** and **108**) prior to the cascade and the addition of cesium fluoride to facilitate the removal of the TMS groups. An increase in efficiency per epoxide was observed when one THP ring in the polyepoxide substrate is formed prior to the cascade. The authors suggest that the cascades proceed as a sequence of silyl-directed epoxide openings by the alcohol nucleophile followed by protiodesilylation, which may occur *via* a homo-Brook rearrangement pathway. After each Brook rearrangement, removal of the silyl group by fluoride reveals the alcohol nucleophile for the next stage of the cascade reaction.

Although disappearing directing groups address problems related to the removal of substituents not present in the natural targets, these reactions suffer from the inability to incorporate the methyl substituents found occasionally at the ring junctions of ladder polyethers.

### 5.2. Reactions of disubstituted epoxides

**Scheme 15:**
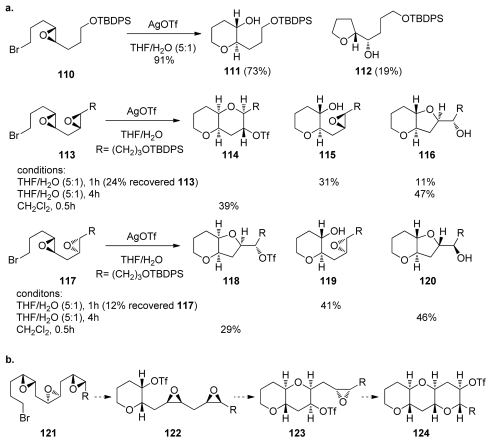
(**a**) Cascades of epoxy halides and polyepoxy halides selectively activated with silver salts. (**b**) Proposed cascade of an all-*cis* polyepoxide, initiated by silver(I) and propagated by the weakly nucleophilic triflate anion [126].

The challenge of achieving high 6-*endo* selectivity in intramolecular epoxide-opening reactions has been approached by several research groups. The first attempt to perform a cascade reaction that does not rely on the effects of directing groups and incorporates only disubstituted epoxides was reported by Murai [126]. The Murai group attempted a conceptually different way of promoting epoxide-opening cascades. They envisioned that the activation of an epoxy halide such as **110** with a silver salt would selectively generate an epoxonium ion at one end of the polyepoxide chain thus ensuring selective activation of a single epoxide in the chain (Scheme 15). This epoxonium ion would then serve as an electrophile for the nucleophilic attack by the neighboring epoxide, thereby forming a new ring and a new epoxonium intermediate and thus propagating the cascade. The direction of the cascade in these reactions is therefore controlled by the position of the halide.

Murai and coworkers first showed that the opening of the epoxonium ion derived from bromo epoxide **110** with an external nucleophile can preferentially provide the desired tetrahydropyran **111** under appropriate conditions (Scheme 15a). However, when more than one epoxide is present in the starting material, the reactions proved to be more capricious, and diverse products from a number of different pathways were formed [126]. Similar investigations on silver-salt promoted cyclizations of polyepoxy bromides by the Jamison group closely mirror the results obtained by Murai [127]. External nucleophiles present in the reaction mixture, such as water, competed with the epoxide oxygen in opening the epoxonium ion. If external nucleophiles were rigorously excluded and the reaction was activated with AgOTf, the triflate anion competes with the epoxide, thus producing yet another electrophilic species that undergoes another displacement reaction to give the *cis* geometry at the ring junction *via* double inversion at the C4 position of **113** and **117** (Scheme 15a). If such a trend were to hold in the case of a polyepoxide, then an all-*cis* polyepoxide could lead to formation of the *trans*-*syn-trans* fragments of ladder polyethers (Scheme 15b). In this scenario, the initial epoxonium ion would be opened by a triflate anion that would, in turn, be displaced by the neighboring epoxide to generate the new epoxonium, thus propagating the cascade.

**Scheme 16:**
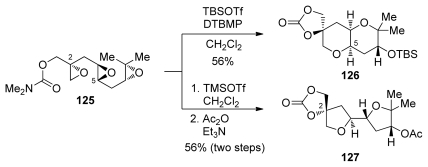
Disubstituted epoxides in silyltriflate-promoted cascades [128]. DTBMP = 2,6-di-*tert*-butyl-4-methylpyridine.

The McDonald group has described efforts to incorporate a disubstituted epoxide in cascade substrates that also contain electronically biased epoxides such as **125** [128]. Aware of the effects that different Lewis acids can have on similar cascade reactions of electronically biased polyepoxides, McDonald and coworkers evaluated various Lewis acids as activators in epoxide-opening cascades of triepoxide **125**. When trimethylsilyl triflate was used with triepoxide **125**, the product of an all-*exo* epoxide-opening cascade **127** was isolated (Scheme 16). The stereochemistry at C2 was preserved in this cascade, suggesting that epoxide opening occurs with retention (or double inversion) at this site. In contrast, when *tert*-butyldimethylsilyl triflate was used, a *syn*-fused THP diad **126** was isolated, suggesting that opening of the epoxonium intermediate proceeds with retention of configuration. Although the reasons for these apparent differences are unclear, it was proposed that the weakly nucleophilic triflate anion may compete with the epoxide oxygen in the opening of the epoxonium intermediate, leading to double inversion and net retention of configuration at C5.

**Figure 7:**
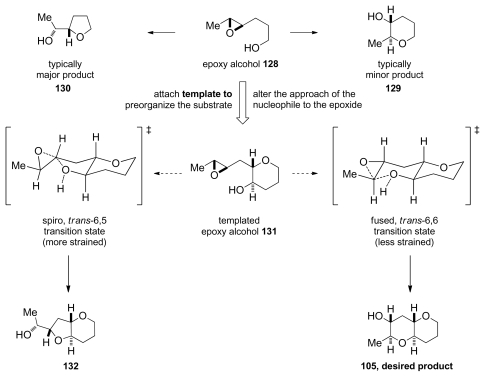
Design of the templated substrates for *endo*-selective epoxide opening [129].

Achieving selective cascade reactions in the absence of any directing group remains a significant challenge, though recently some progress in this area has been made. Although 6-*endo*-cyclization of simple epoxy-alcohols is kinetically disfavored, the Jamison group has explored the use of substrate control to reverse the selectivity of these reactions [129]. The Jamison group reasoned that pre-organization of the substrate in an appropriate fashion, perhaps by forming one THP ring before the cyclization (as in **104**, Scheme 14), could encourage the cyclization towards the *endo* pathway by altering the approach of the alcohol nucleophile to the epoxide in **131** (Figure 7). *Trans-*bicyclo[ 4.4.0]decane derivatives are typically less strained than their *trans*-bicyclo[4.3.0]nonane counterparts. If the greater stability of the *endo*-product **105** compared to the *exo*-product **132** could be reflected in the transition states, it would translate into a kinetic selectivity for the desired *trans*-fused polytetrahydropyrans.

**Figure 8:**
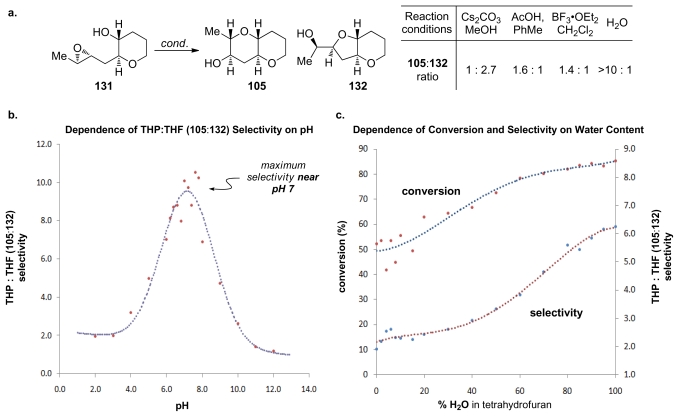
(**a**) Epoxide-opening reactions of templated epoxy alcohol **131**. (**b**) Dependence of selectivity in epoxyalcohol cyclizations on the pH of the reaction medium. (**c**) The effect of water on conversion and selectivity of epoxyalcohol cyclizations in THF/water mixtures [129].

During their investigation of this hypothesis, Jamison and coworkers discovered that regioselectivity in cyclization reactions of the templated epoxy alcohol **131** were significantly improved compared to the generally observed selectivities for simple epoxy alcohols (approx. 1:6 favoring undesired product) [129] (Figure 8a). The dependence of regioselectivity on the reaction conditions was also noted. While the *exo*-product **132** is favored under basic conditions (Cs_2_CO_3_ in MeOH) with selectivities of about 1:3, under both Lewis and Brønsted acidic conditions a slight preference for the larger THP-containing product **105** was observed (up to 1.6:1). Exploration of the reactions in aqueous media, where buffers allow for careful control of acid and base concentrations, revealed an interesting trend. The selectivity for the desired THP product **105** increases substantially as the pH of the reaction environment approaches neutrality (Figure 8b,c) independent of the buffer identity. Furthermore, simple deionized water promotes cyclization of **131** with greater than 10:1 *endo:exo* selectivity, thus revealing water as the key promoter in these reactions.

**Figure 9:**
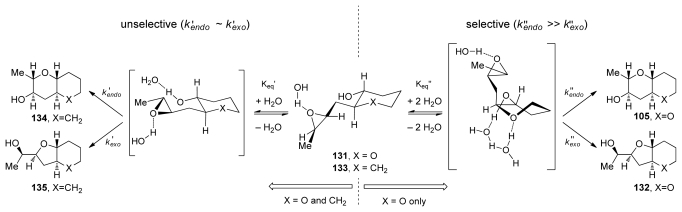
Mechanistic rationale for *endo* selectivity in cyclizations of templated epoxy alcohols in water (although transition state of the selective pathway is depicted with three water molecules, it is possible that one of these molecules originates from the solvated ground state) [130].

Hydrogen bond interactions between the THP template, epoxide and water molecules were proposed as the origin of *endo* selectivity in described reactions, though these effects were not well understood. Kinetic studies on **131** and the related epoxy alcohol **133**, with a cyclohexane in place of the THP template, were performed in order to probe the possible mechanistic origins of previously reported observations [130]. The Jamison group disclosed evidence that suggest existence of the competing mechanisms that are first and second order in water (Figure 9). The pathway which is second order in water is selective and operates only for the epoxy alcohol with a THP template. The other pathway is unselective. Jamison hypothesized that epoxy alcohol cyclizations in water occur for hydrated conformations that possess the appropriate geometry for the reaction. The presence of the oxygen in the template of **131** likely serves two functions. First, its inductive electron-withdrawing effect electronically biases the epoxy alcohol towards *endo* cyclization both by decreasing the nucleophilicity of the alcohol and by discouraging the development of positive charge at the nearby *exo* site of attack on the epoxide. Second, the THP oxygen may also facilitate reaction through a putative *endo*-selective pathway that is second order in water, possibly *via* an intermediate with the tetrahydropyran template in a twist-boat conformation (Figure 9).

Templated epoxide-opening cascade reactions promoted by water were then examined with diepoxide **136** and triepoxide **137**, where all epoxides lack directing groups [129]. Both cascades proceed in good yield in water at elevated temperatures, affording the THP triad and tetrad subunits **100** and **138** that are found in more than half of the known ladder polyether natural products. Interestingly, diepoxide **136** fails to cyclize to the THP triad **100** under basic conditions (Cs_2_CO_3_ in MeOH) and affords the desired product in only trace quantities under both Lewis and Brønsted acidic conditions (CSA or BF_3_·Et_2_O in CH_2_Cl_2_).

**Scheme 18:**
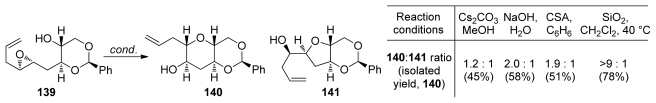
Cyclization of benzylidene acetal-templated epoxy alcohols [131].

With the goal of applying directing group-free epoxide-opening cascades in water towards the synthesis of ladder polyether fragments, Jamison investigated other oxygen-containing templates that would produce synthetically useful fragments primed for fragment coupling or further elaboration to ladder polyether natural products [131]. Evaluation of the benzylidene acetal template revealed some differences in the reactivity of epoxy alcohols featuring this template compared to those with the THP template (Scheme 18). Due to the slow cyclization rates in water, the benzylidene acetal templated epoxy alcohol **139** requires extended reaction times at elevated temperatures that results in competitive acetal hydrolysis. Interestingly, silica gel and its hydrated form, silicic acid, in dichloromethane serve as excellent promoters in the cyclization reactions of epoxy alcohol **139** that proceed with greater than 9:1 *endo* selectivity, affording the bicycle **140** in good yield. Silica, like water, can serve as both hydrogen bond donor and hydrogen bond acceptor and may thereby facilitate proton transfer during cyclization of the epoxy alcohol. Silica and other amphoteric oxides similar to silica may represent a flexible and valuable scaffold for the design of new cyclization promoters.

**Scheme 19:**
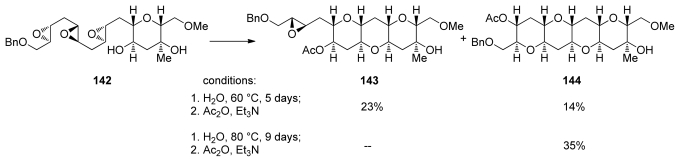
Application of epoxide-opening cascades promoted by water in the synthesis of HIJK fragment of gymnocin A [131].

Upon silica promoted *endo*-cyclization, **140** was elaborated into the triepoxy alcohol **142** that features a highly functionalized THP template. Incubation of **142** in water at 60 °C for 5 days followed by acetylation afforded some of the desired THP tetrad **144** and a larger quantity of **143** in which two THP rings had formed but the final epoxide remained intact (Scheme 19). A higher reaction temperature and longer reaction time (80 °C, 9 days) drove the reaction to completion and subsequent acetylation allowed the isolation of **144**, the HIJK fragment of gymnocin A in 35% yield. THP tetrad **144** bears four differently substituted pendent hydroxyl groups ready for synthetic elaboration [131].

**Scheme 20:**
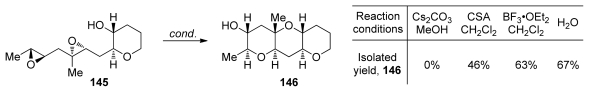
Epoxide-opening cascades of substrates with disubstituted and trisubstituted epoxides [111].

The described directing group-free epoxide-opening cascades successfully produce polyether fragments without methyl substituents at the ring junctions. To extend the utility of this work, the Jamison group demonstrated that these aqueous cascades tolerate the presence of methyl groups at positions of desired 6-*endo* nucleophilic attack in which their directing effects promote the desired cascade outcome [110,111]. Under aqueous conditions, substrate **145** which incorporates both a trisubstituted and a disubstituted epoxide affords the triad **146** (Scheme 20). This reaction overcomes the need for alkyl substituents to be uniformly distributed on each epoxide as in previously described methyl-directed cascades. Notably, diepoxide **145** fails to produce any of the triad **146** under basic conditions and affords the desired product in lower yield under both Lewis and Brønsted acidic conditions.

As noted earlier, methyl groups are typically randomly distributed across the ring junctions of the ladder polyether natural products. Thus, putative polyepoxide precursors of ladder polyethers often feature two distinctly different types of trisubstituted epoxides along with the disubstituted epoxides. Methyl groups of trisubstituted epoxides can be situated in positions where their directing effect will promote *endo*-opening under acidic conditions (as in **145**), or alternatively in positions that would normally promote *exo*-selective epoxide-opening under the same conditions (as in **150**). For this reason, a general method for synthesis of polytetrahydropyran fragments of ladder polyethers would be required to accommodate all three varieties of epoxide substitution. In other words, it must overcome any adverse directing effects of alkyl groups.

**Scheme 21:**
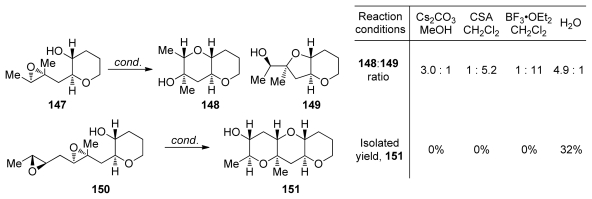
Water overcomes methyl group directing effects in epoxide-opening cascades [111].

Under strongly basic conditions nucleophiles normally, but not without exceptions, open the less substituted site on the epoxide. However, these conditions are not suitable for epoxide-opening cascades. Jamison disclosed the first example of 6-*endo* epoxide-opening cyclization on trialkyl epoxide that overcomes the directing effect of alkyl group under mild conditions [111]. Cyclization of templated epoxide **147**, perhaps not surprisingly, yields *endo*-product **148** under basic conditions and *exo*-product **149** when reaction is promoted with acid (Scheme 21). In water, the larger THP product is favored with selectivity of about 5:1. Water, in combination with the THP template, also proved successful in controlling the regioselectivity in epoxide-opening cascade leading to the methylated THP triad **151** by overturning the directing effect of the methyl substituent in diepoxide **150** (Scheme 21). A number of different reaction conditions were evaluated for the cyclization of diepoxide **150** but only cascades in water provided any of the THP triad **151**. The transformation of **150** to **151** represents the first *endo*-selective epoxide-opening cascade to accommodate this type of methyl substitution.

## 6. Construction of Oxepanes *via Endo*-Selective Epoxide-Opening Reactions

All ladder polyether natural products and all oxasqualenoids with fused rings feature medium sized cyclic ethers in their structures. As discussed before, tetrahydropyrans are proposed to be constructed *via endo*-selective epoxide-opening reactions in the producing organisms. Larger rings are proposed to arise in processes analogous to construction of THPs. The challenges related to construction of THP rings *via* intramolecular *endo*-cyclization of 4,5-epoxy alcohols were discussed above. Although THP rings are thermodynamically favored due to a minimum of ring strain, smaller THF rings are normally formed in these reactions as a consequence of a kinetic preference for the transition state leading to the smaller ring product. Similar analysis of 5,6-epoxy alcohols that would produce oxepane rings *via endo*-cyclizations reveals additional challenges that this process presents. As in the previous case, smaller tetrahydropyran products are kinetically favored. Unlike the formation of THPs *via endo* epoxide opening where the larger ring is thermodynamically favored, construction of oxepanes *via endo*-selective epoxide opening is further plagued by the thermodynamic preference for the smaller ring, which lacks ring strain. In short, for unbiased systems, the 7-*endo* pathway is both kinetically and thermodynamically disfavored. For this reason, almost all *endo*-selective epoxide-opening reactions that produce oxepane rings depend upon electronically biased epoxides.

### 6.1. Reactions of electronically biased epoxides

Many of the strategies to bias the epoxide electronically towards *endo*-opening in the synthesis of THPs have also been applied to the corresponding substrates primed for the synthesis of oxepanes *via endo*-selective epoxide-opening reactions.

**Scheme 22:**
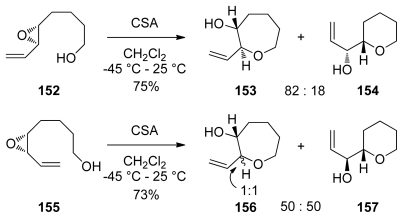
Activation of 7-*endo* over 6-*exo* epoxide opening: an alkenylepoxide approach to the synthesis of oxepanes *via* epoxide opening [132].

Acid-catalyzed cyclization reactions of alkenyl epoxides in which 7-membered rings are produced have been reported by the Nicolaou group [132]. As in related 6-*endo*-selective reactions, an electron-rich alkenyl substituent is required for good selectivity. However, selectivities observed in formation of oxepanes are much lower than was observed for tetrahydropyrans (Scheme 22). In addition to lower selectivities, some of these reactions do not proceed with clean inversion of configuration at the site of nucleophilic attack, perhaps due to the slower rates of cyclization. Subsequent studies by the Nakata and Mukai groups echoed the limitations described by Nicolaou. While reactions of the styryl epoxides studied by Nakata and coworkers proceed with improved *endo* selectivity than those of the alkenyl substrates reported by Nicolaou, epimerization of the epoxide stereocenter and isomerization of the styryl double bond are more pronounced for these substrates [133]. The same trend was established by Mukai for Lewis acid-catalyzed reactions of 5,6-epoxy-7-octyn-1-ols and their cobalt complexes [134,135].

**Scheme 23:**
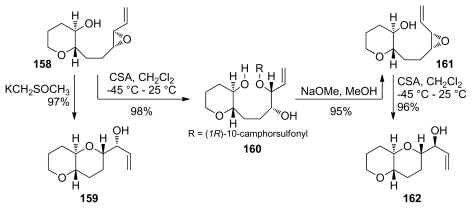
Alkenylepoxides fail to produce oxepanes in the fused THP/oxepane bicyclic systems [132].

Cyclization reactions of the alkenyl epoxides **158** and **161** in which one THP ring is already formed fail to produce the desired fused THP/oxepane bicycle under a variety of conditions [132]. If acidic promoters such as CSA are used, the nucleophilic attack of the conjugate base outcompetes the *endo* cyclization pathway (Scheme 23). This result is the direct consequence of the slow rate of 7-*endo* cyclizations. Reports of the application of *endo*-opening of alkenyl epoxides in synthesis of the oxepane rings in ladder polyethers are thus scarce.

**Scheme 24:**
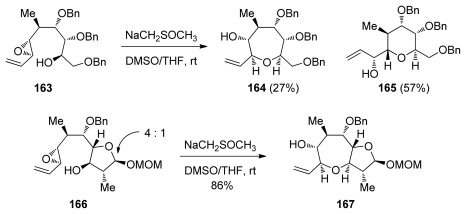
Cyclization of alkenylepoxides to form the K ring of ciguatoxin CTX1B [136].

The groups of Sasaki and Tachibana have utilized this methodology to construct the highly substituted K-ring of ciguatoxin CTX1B [136] (Scheme 24). Interestingly, an open chain substrate **163** undergoes cyclization under acidic conditions to produce a 1:1 mixture of a THP and THF products that arise *via endo*-opening by the oxygen from benzyl ethers at the C3 and C4 positions, respectively. Treatment of **163** with dimsyl base, however, forms some of the oxepane, albeit with low selectivity (approx. 1:2 favoring the *exo*-product, Scheme 24). In contrast to previous work by Nicolaou [132], when one ring is already in place as the mixed acetal in the epoxy alcohol **166**, the cyclization reaction proceeds with good selectivity to afford the desired bicyclic system. It was noted that the regioselectivity in these reactions depends on the configuration of the mixed acetal.

**Scheme 25:**
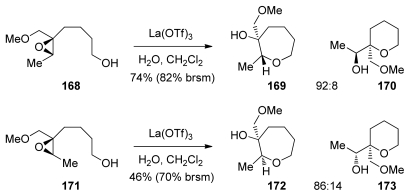
La(OTf)_3_-catalyzed 7-*endo*-selective cyclizations of methoxymethyl substituted epoxy alcohols [137].

Methoxymethyl substituents were previously shown to direct 6-*endo*-cyclization in combination with lanthanide Lewis acids. Extension of this work to the corresponding substrates primed for 7-*endo* cyclization was reported by Murai [137]. These reactions also require a specific set of conditions: use of stoichiometric chelating lanthanum(III) trifluoromethylsulfonate as a Lewis acid and 3.3 equivalents of water in dichloromethane (Scheme 25). The reactions proceed significantly slower than the corresponding 6-*endo*-selective reactions. Examples of epoxide-opening cascades to form oligooxepanes were not reported.

**Scheme 26:**
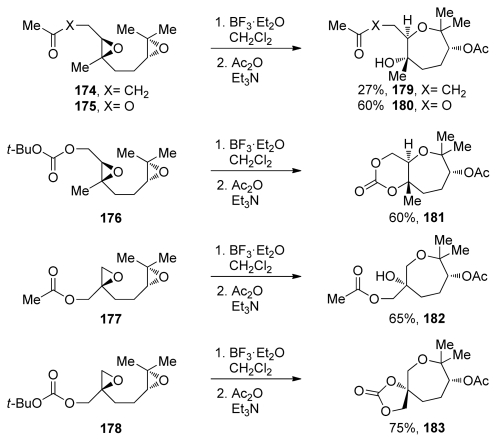
Lewis acid-catalyzed epoxide-opening cascades that form oxepanes from 1,5-diepoxides [142,143].

Other than alkenyl and methoxymethyl substituted epoxides, most of the work on 7-*endo*-selective epoxide opening has been done on trisubstituted epoxides featuring a directing methyl group. Early examples have shown that acid-catalyzed cyclizations of appropriately substituted trialkyl epoxides can produce oxepanes in good yield [138–141]. The systematic studies of directing effects of methyl substituents and their application in the synthesis of polyoxepanes *via* epoxide-opening cascades were reported by the McDonald group [142,143]. McDonald and coworkers examined cascade reactions that include the formation of an oxepane ring and *trans-*fused bisoxepane motifs *via endo*-selective epoxide openings (Scheme 26). Effects of the terminating nucleophile were also evaluated as was previously described for smaller ring systems.

**Scheme 27:**
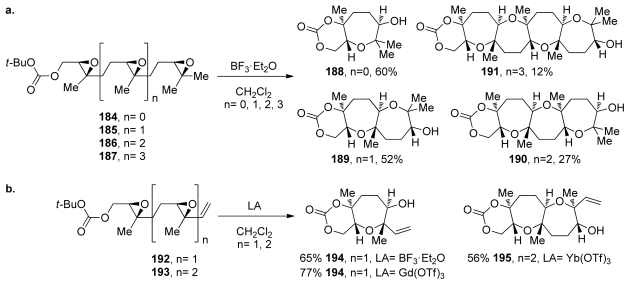
(**a**) Methyl-directed epoxide-opening cascades forming polyoxepane systems. (**b**) Lewis acid-promoted cyclizations of polyepoxide substrates with terminal alkenyl epoxides [107,144].

The McDonald group extended this approach to polyepoxides for the synthesis of polyoxepane systems **184**–**187** [107,144]. The efficiency of these reactions decreases as the number of epoxides in the polyepoxide precursor increases (Scheme 27a). Unselective activation of any of the epoxides in the starting materials may play a central role in lowering the efficiency of these cascades [107]. If the selective activation of only the epoxide that is distal to the terminating nucleophile could be achieved, the cascades might proceed in one direction exclusively, and higher yields should be observed. In attempts to achieve this, the McDonald group prepared substrates **192** and **193**, which feature a vinyl and methyl substituent instead of the two geminal methyl substituents on the terminal epoxides in the polyepoxide chains of **184**–**187** (Scheme 27b). Based on previous work on alkenyl epoxides, it was expected that the stabilization provided by the vinyl substituent would not only improve the selectivity in epoxide-opening reactions, but also lead to selective activation of the alkenyl epoxide over the interior epoxides. The desired oxepane ring-containing products **194** and **195** were produced in yields that are slightly higher than in the corresponding reactions of substrates **184** and **185** that lack vinyl substituents [144]. Gd(OTf)_3_ and Yb(OTf)_3_ proved to be the most efficient Lewis acid promoters for these cascades. It is worth noting that these cascades are tolerant of epoxysilanes. Work on an analogue of **193** in which the methyl group is replaced with trimethylsilyl substituent revealed that the cascade reaction proceeds with just slightly lower efficiency.

**Scheme 28:**
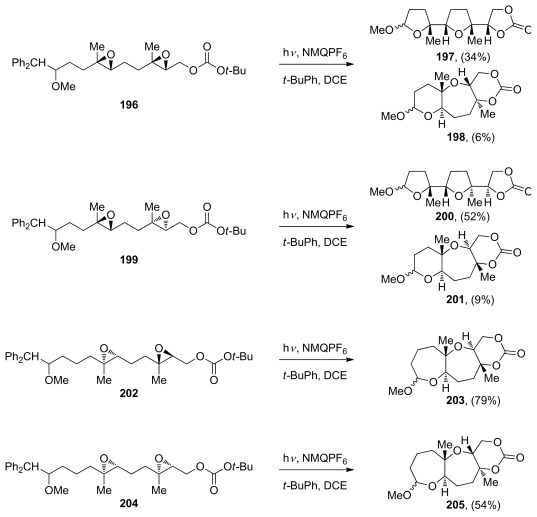
Epoxide-opening cascades initiated by oxidative cleavage of homobenzylic ethers [115]. NMQ = *N*-methylquinolinium; DCE = 1,2-dichloroethane.

The concept of the selective generation of a reactive epoxonium intermediate on one end of a polyepoxide substrate was introduced by the Murai group in their work toward the synthesis of ladder-type polyethers (*vide supra*, Scheme 15). Floreancig and coworkers have demonstrated that single-electron oxidation is another effective method for the selective initiation of an epoxide-opening cascade [145,146]. They use mesolytic carbon-carbon bond cleavage in the benzylic position of the radical cations of homobenzylic ethers (such as **196**, **199**, **202** or **204**), to form oxonium ions that react with pendent epoxides thus producing epoxonium ions, which can then undergo further cyclization (Scheme 28). The Floreancig group, in collaboration with Houk and coworkers, published experimental and computational studies on the structure-reactivity relationships for intramolecular additions to bicyclic epoxonium ions [115]. They observed that ring size has a significant impact on these processes, with *endo*-cyclizations being preferred for bicyclo[4.1.0] epoxonium ions bearing an alkyl directing group, and *exo*-cyclizations being preferred for bicyclo[3.1.0] epoxonium ions despite the presence of a directing group (Scheme 28). The authors propose that these effects can be attributed to the ability of the larger ring to accommodate a looser transition state with significant S_N_1 character, thereby promoting the *endo* process regardless of solvent polarity. Knowing that the epoxonium ion structure is a significant determinant of regioselectivity under these kinetic cyclization conditions, Floreancig and coworkers then designed a number of extended substrates that undergo cascade cyclizations to form fused tricyclic systems under the same oxidative conditions (Scheme 28).

**Scheme 29:**
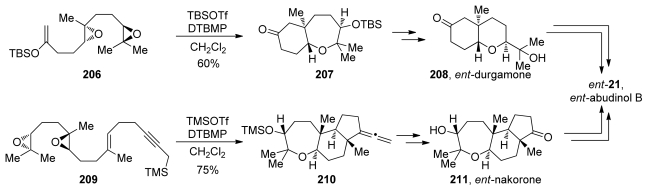
Convergent synthesis of *ent*-abudinol *via* hybrid oxa/carbacyclization [147].

Evaluation of the directing effects of methyl groups in epoxide-opening cascades first came to fruition in the elegant syntheses of the triterpenoid *ent*-abudinol B and the related terpenes *ent*-durgamone and *ent*-nakorone [147]. While these products are not polyethers, the oxa/carbacyclization approach taken by McDonald is closely related to the epoxide-opening cascades previously reported by his group. In their first-generation approach to *ent*-abudinol, McDonald and coworkers devised a convergent synthetic scheme that involves late-stage coupling of fragments derived from *ent*-durgamone and *ent*-nakorone (Scheme 29). In the synthesis of subunit **208**, a cascade of epoxide openings of diepoxide **206** was employed. Using *tert*-butyldimethylsilyl triflate as a Lewis acid, two *endo*-selective cyclizations directed by methyl substituents with an enolsilane as trapping nucleophile, leads to formation of bicyclic compound **207**, which can be further elaborated to **208**, *ent-*durgamone. An analogous strategy was utilized in the synthesis of the more complex *ent*-nakorone (Scheme 29). Along with epoxide openings, a hybrid cascade of oxacyclizations and carbacyclizations was designed. Diepoxide **209**, which carries a terminating propargyl silane nucleophile, underwent efficient trimethylsilyl triflate-promoted cyclization, resulting in the formation of the tricyclic allene **210**. Further elaboration of these fragments into their corresponding vinyl triflates and subsequent modified Suzuki-Miyaura coupling produced *ent*-abudinol B.

**Scheme 30:**
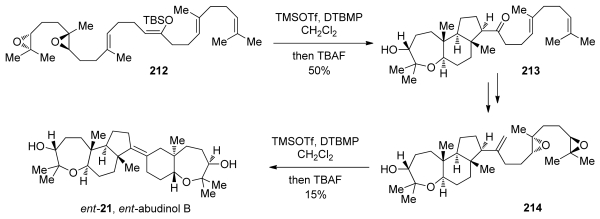
Biomimetic synthesis of *ent*-abudinol B [148]. TBA = tetrabutylammonium.

A second-generation approach was based on the proposed biosynthetic pathway to *ent*-abudinol, which involves a hybrid cascade of epoxide openings and carbacyclizations [148]. In a fashion similar to the first-generation approach, diepoxide **212** was treated with trimethylsilyl triflate to produce **213**, which contains the tricyclic fragment of *ent*-abudinol (Scheme 30). A two-step elaboration of the cascade product **213**, using a Wittig methylenation and Shi epoxidation, resulted in the formation of diepoxide **214**, thus setting the stage for a cascade reminiscent of that used for *ent*-durgamone. Diepoxide **214**, which carries a terminal alkene instead of an enol-ether as the trapping nucleophile, was subjected to the same conditions as in the reaction of **209** to **210** to produce *ent*-abudinol, along with several isomeric products resulting from pathways enabled by the relatively low nucleophilicity of the terminating alkene (Scheme 30). Despite the linear nature of this route to *ent*-abudinol, structural complexity is generated quickly, and this rapid synthesis demonstrates the advantages of cascade approaches to the synthesis of polyethers and related molecules. McDonald and coworkers have also prepared (3*R*,6*R*,7*R*,18*R*,19*R*,22*R*)-squalene tetraepoxide, a putative biosynthetic precursor to a variety of oxasqualenoids including abudinol B. This intermediate, however, failed to cyclize to *ent*-abudinol B [149].

**Scheme 31:**
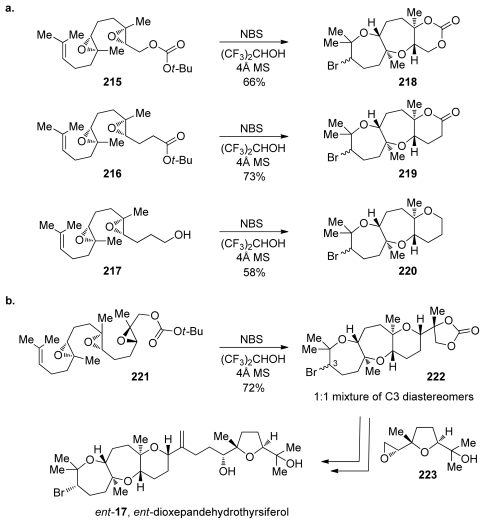
(**a**) Bromonium-initiated epoxide-opening cascades. (**b**) Total synthesis of *ent*-dioxepandehydrothyrsiferol [60]. NBS = *N*-bromosuccinimide.

Jamison and coworkers reported the first synthesis of a natural product featuring a *trans*-*anti*-*trans* tricyclic polyether, oxasqualenoid *ent*-dioxepandehydrothyrsiferol [60]. Their approach relies on a well-documented bromoetherification strategy for formation of the bromooxepane [118,150]. However, Jamison and coworkers extended the utility of this reaction, implementing it in epoxide-opening cascades on diepoxides **215**–**217** and ultimately triepoxide **221** that forms the tricycle of *ent*-dioxepandehydrothyrsiferol (Scheme 31). Initiation of the cascades *via* the electrophilic activation of an alkene using a bromonium source secures good control over the directionality. Regioselectivity of the epoxide openings is controlled by the presence of methyl groups at each of the epoxides. The reactions proceed best in the highly polar non-nucleophilic solvent hexafluoro-*iso*-propanol, likely by facilitating a cationic cascade that maximizes the directing influence of the methyl groups. Complete inversion of configuration in each of the epoxide openings allows for the geometry of the ring junctions to be controlled by the stereochemical composition of the starting polyepoxides. Upon treatment of **221** with NBS in hexafluoro-*iso*-propanol, the cascade proceeded with the predicted regioselectivity in the bromonium-opening and all epoxide-opening events, furnishing the desired tetracycle **222** in good yield as a 1:1 mixture of diastereomers resulting from unselective bromonium formation (Scheme 31b).

### 6.2. Reactions of disubstituted epoxides

The examples outlined in the first part of this chapter clearly demonstrate that 7-*endo*-cyclizations to produce oxepanes are often difficult and quite challenging even with electronically biased epoxides. Incorporation of the directing groups at every ring junction of the final cascade product renders these otherwise impressive cascades hard to apply in synthesis of ladder polyether fragments. Were these cascades to be used in the synthesis of naturally occurring molecules, they would have to accommodate polyepoxides without directing groups (disubstituted epoxides) and allow for a variety of substitution patterns that would install methyl groups only at the desired positions of the final products. However, the removal of all directing groups renders the task of achieving 7-*endo-*cyclizations quite daunting due to the very limited opportunities for control of regioselectivity in these reactions. Since 7-*endo* pathways in simple epoxy alcohols are both kinetically and thermodynamically disfavored, a logical choice would be to apply a reaction with reagent control of regioselectivity. However, such reagents have yet to be identified. This is part of the reason why reports of 7-*endo-*selective ring-closures onto disubstituted epoxides are extremely scarce and highly dependent on the substrate structure.

**Scheme 32:**
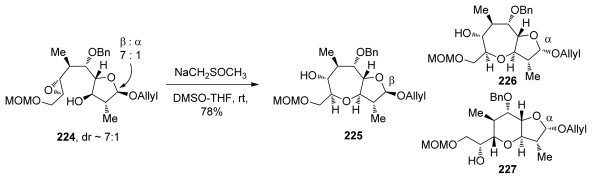
*Endo*-selective opening of a disubstituted epoxide in the synthesis of K ring of ciguatoxin CTX1B (regioselectivity of epoxide opening depends on the configuration of mixed acetal) [136].

The finding that the cyclization of **163** under acidic conditions fails to preferentially produce the oxepane ring, while strong dimsyl base enables this transformation to proceed with ease and in good selectivity (*vide supra*, Scheme 24) prompted the groups of Sasaki and Tachibana to attempt analogous transformations on the related epoxy alcohol **224** that lacks an alkenyl substituent on the epoxide (Scheme 32) [136]. In fact, epoxy alcohol **224** may be considered to be electronically biased towards the 6-*exo* pathway due to the electron withdrawing effects of the MOM ether attached to the distal position on the epoxide. Somewhat surprisingly, cyclization of **224** under basic conditions proceeds with good selectivity to afford oxepane **225**. It is interesting to note that the configuration of the hemiacetal plays an important role in determining the regioselectivity of the reaction. While the β-anomer yields the *endo* product **225** exclusively, regioselectivity in cyclization of the α-anomer is significantly lower (about 1.7:1 favoring the larger ring **226** over **227**). Sasaki and Tachibana suggest that the structure of the substrate is responsible for the high *endo* selectivity in these reactions. However, an elaborate analysis of specific requirements to achieve the *endo* epoxide opening in other systems was not provided.

**Scheme 33:**
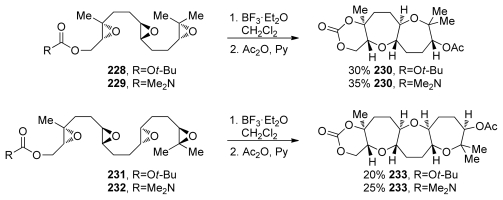
Lewis acid-catalyzed synthesis of polyoxepanes from polyepoxides featuring unbiased, disubstituted epoxides [151].

The McDonald group also investigated cascades that incorporate disubstituted epoxides as in substrates **228**, **229**, **231** and **232** [151] (Scheme 33). The difference between the electronic properties of disubstituted and trisubstituted epoxides may work in favor of the desired, Lewis acid-initiated cascade through preferential activation of the epoxide distal to the terminating nucleophile (as the most electron rich epoxide in the chain). This is conceptually similar to the cascades on alkenyl polyepoxides **192** and **193** (*vide supra*, Scheme 27b). Cascades of both triepoxides **228** and **229** and tetraepoxides **231** and **232** proceed under standard Lewis acid activation to form the desired tricyclic (**230**) and tetracyclic (**233**) polyethers (Scheme 33). It was proposed that once the first epoxonium ion is formed at the distal end, the transition states leading to *endo* and *exo* opening of the disubstituted epoxonium ion differ in energy, with a higher degree of ring strain associated with the bicyclo[3.1.0] intermediate than for the bicyclo[4.1.0] intermediate formed as the product of *endo* opening. The authors also noted that a directing group is required on the epoxide proximal to the trapping nucleophile, as there is minimal strain associated with either the 5- or 6-membered carbonates formed at the end of the cascade if carbonate or carbamate nucleophiles are used. A directing group is therefore necessary to ensure *endo* regioselectivity in the opening of this last epoxide.

Similar to the work of McDonald, the Floreancig group successfully incorporated a disubstituted epoxide in their cascades initiated *via* a single-electron oxidation of homobenzylic ethers [115]. The yields and selectivity for the desired fused products are also lower compared to substrates with directing groups.

## 7. Future Directions

Despite the large body of work toward the development of *endo*-selective epoxide-opening cascades capable of producing larger fragments of ladder polyethers in a single synthetic operation, this goal remains elusive. Many of the challenges that such cascades present have been overcome through the creative efforts of synthetic chemists. Yet all of the epoxide-opening cascades developed to date have limitations that prevent them from fully emulating the biosynthetic proposal for these compounds.

*Endo*-cyclizations onto disubstituted epoxides to produce medium-sized cyclic ethers (7-, 8- or 9- membered) remain a major challenge. This is also true for epoxide-opening cascades that incorporate these reactions as a discrete step. Only a few reactions successfully construct oxepanes without the use of directing groups attached to the epoxide and not without imposing rigid limitations on the structure of the products. Data on *endo* epoxide cyclizations to produce oxacanes (8-membered rings) is extremely limited. Furthermore, nine-membered rings have never been produced *via endo*-opening of an epoxide. Finally, with the exception of the cascade used in the recent total synthesis of *ent*-dioxepandehydrothyrsiferol, successful *endo*-selective epoxide-opening cascades produce only cyclic ethers of one ring size, either polytetrahydropyrans or polyoxepanes *via* epoxide openings. Designing a single cascade that produces fused cyclic ethers of various sizes is still a challenge.

The longest cascade reported to date constructs four fused cyclic ethers in a single operation (*vide supra*, Scheme 27a). The efficiency of these cascade reactions decreases significantly as the number of epoxides in cascade substrates increases. Activation of an epoxide for attack by nucleophile necessarily makes the epoxide susceptible to attack by neighboring epoxides in the cascade substrates. Hence, the larger number of epoxides in the cascade substrate leads to increased production of side products. Circumventing this problem would, in theory, enable the synthesis of larger fragments of natural products *via* cascade reactions with a larger number of epoxides.

Development of reagents to control the regioselectivity of epoxide-opening reactions may hold promise for future advancement of the field. There are several avenues that lead to such goal. Developing small molecule activators or catalysts for *endo*-selective epoxide opening would be one possible approach. Another is the identification and isolation of epoxide-hydrolase enzymes that may be involved in the biosynthesis of ladder polyethers.

**Scheme 35:**
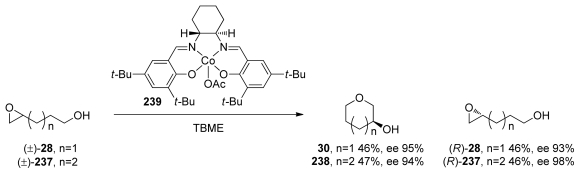
Regio- and enantioselective cyclization of epoxy alcohols catalyzed by a [Co^III^(salen)] [152,153]. TBME = *tert*-butylmethylether.

Several reports demonstrate that the control of selectivity in epoxide-opening reactions by small molecules and proteins is possible and suggest that research in both of these areas may prove worthwhile. For example, the Jacobsen group successfully applied a [Co^III^(salen)] complex **239** to the kinetic resolution of 4,5- and 5,6-epoxy alcohols (**28** and **237**, Scheme 35) [152,153]. Kinetic resolution in these reactions is achieved *via* selective cyclization of one enantiomer of **28** or **237**. This cyclization appears to be completely *endo*-selective. The groups of Parquette and RajanBabu recently reported related reactions in which yttrium-salen complexes enable divergent regioselectivity in ring opening reactions of chiral aziridines [154], thus demonstrating the generality of this approach.

The ability of proteins to promote *endo*-selective intramolecular epoxide opening and govern regioselectivity was first demonstrated by Janda and coworkers [113,155,156]. They were able to generate and isolate a catalytic antibody that promoted 6-*endo* epoxide opening of epoxy alcohol **240** (Scheme 35). As expected, catalytic antibody 26D9 also performs a kinetic resolution, transforming only one of the enantiomers of the substrate to the corresponding tetrahydropyran **241** [113,155].

**Scheme 37:**
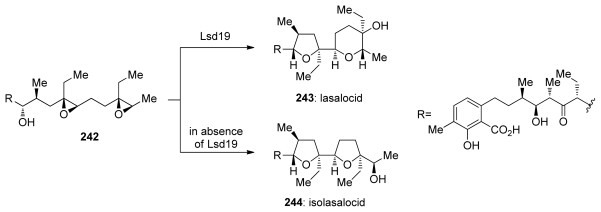
Lsd19 catalyzes *endo* epoxide opening in biosynthesis of lasalocid A [158].

While the work of Leadlay on the biosynthesis of monensin A provided substantial evidence in favor of the Cane-Celmer-Westley proposal [157], final, direct evidence for the involvement of an epoxide hydrolase enzyme in the cascade leading to a polyether ionophore lasalocid A was provided only recently by Oikawa and coworkers [158]. They identified *lsd19* among the lasalocid biosynthetic genes in *Streptomyces lasaliensis* as coding the putative epoxide hydrolase. Cloning and expression of *lsd19* in *Escherichia coli* provided epoxide hydrolase Lsd19 in nearly pure form. This enzyme efficiently transforms synthetic prelasalocid diepoxide **242** to lasalocid A *in vitro* (Scheme 37). These studies unambiguously demonstrated that Lsd19 is responsible for the regio- and stereochemical outcome of this epoxide-opening cascade. Most importantly, Lsd19 promotes *endo*-selective epoxide opening to form the THP ring of **243**, lasalocid A. Isolasalocid **244**, an analogue with a THF ring in place of the THP ring of lasalocid A, is isolated in the absence of Lsd19. Concurrent *in vivo* studies by Leadlay and coworkers on the targeted deletion of the gene for Lsd19 provided the final, direct evidence for the role of Lsd19 in directing the regioselectivity of THP formation from the epoxide precursor during the biosynthesis of lasalocid A. Experiments on the producing organism, *Streptomyces lasaliensis* and a mutant that lacks *lasB* (*lsd19*) established that the absence of Lsd19 in the Δ*lasB* mutant causes a complete switch from the production of lasalocid A in *S. lasaliensis* to the production of isolasalocid in the Δ*lasB* mutant [159].

With many challenges remaining in the field of epoxide-opening cascades and exciting opportunities in related areas that have not been extensively researched, future progress can be foreseen. The investigations of epoxide-opening cascades in synthesis and those that are proposed to happen in nature will require collaborative efforts of chemists and biologists.

## Figures and Tables

**Figure 6:**
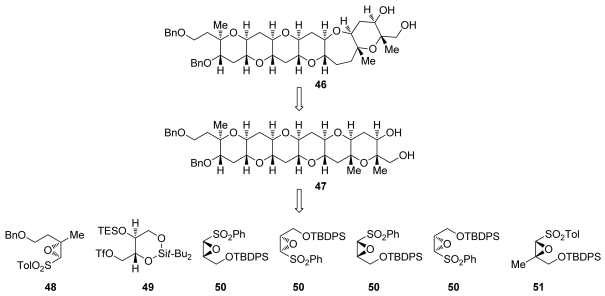
Application of 6-*endo* cyclization of epoxysulfones: conceptual retrosynthesis of the ABCDEF ring fragment of yessotoxin and adriatoxin [102]. Tol = *p*-tolyl; TES = triethylsilyl.

**Scheme 17:**
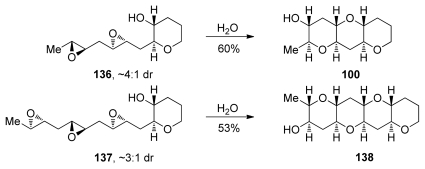
Epoxide-opening cascades promoted by water [129].

**Scheme 34:**
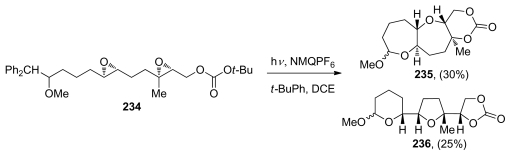
Oxidatively initiated cyclization to polyoxepanes from polyepoxides featuring unbiased, disubstituted epoxide [115].

**Scheme 36:**
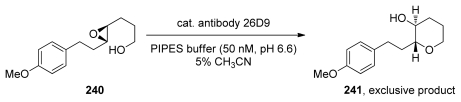
Antibody catalysis of 6-endo epoxide opening [113,155]. PIPES = piperazine-1,4-bis(2-ethanesulfonic acid).

**Table 1:** Effects of the terminating nucleophile on the outcome of methyl-directed cascades [107].

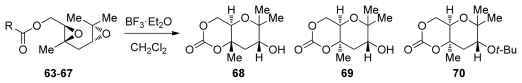							
Entry	Substrate	R	Concentration	T (°C)	Yield of 68 (%)	Yield of 69 (%)	Yield of 70 (%)
1	**63**	*t-*BuO	0.05 M	−40	<4	56	12
2	**63**	*t-*BuO	0.05 M	40	–	65	4
3	**63**	*t-*BuO	0.5 M	−40	–	42	10
4	**64**	PhNH	0.05 M	−40	–	70	–
5	**65**	Me_2_N	0.05 M	−40	35	10	–
6	**65**	Me_2_N	0.05 M	20	55	21	–
7	**66**	(CH_2_)_4_N	0.05 M	−40	32	9	–
8	**67**	O(CH_2_CH_2_)_2_N	0.05 M	−40	34	13	–
